# *Limosilactobacillus reuteri* normalizes blood–brain barrier dysfunction and neurodevelopment deficits associated with prenatal exposure to lipopolysaccharide

**DOI:** 10.1080/19490976.2023.2178800

**Published:** 2023-02-17

**Authors:** Jing Lu, Xiaobing Fan, Lei Lu, Yueyue Yu, Erica Markiewicz, Jessica C. Little, Ashley M. Sidebottom, Erika C. Claud

**Affiliations:** a Department of Pediatrics, The University of Chicago, Pritzker School of Medicine, Chicago, IL, USA; b Magnetic Resonance Imaging and Spectroscopy Laboratory, The University of Chicago, Department of Radiology, Chicago, IL, USA; c Duchossois Family Institute, The University of Chicago, Host-Microbe Metabolomics Facility, Chicago, IL, USA

**Keywords:** Maternal inflammation, lipopolysaccharide, probiotics, blood–brain barrier

## Abstract

Maternal immune activation (MIA) derived from late gestational infection such as seen in chorioamnionitis poses a significantly increased risk for neurodevelopmental deficits in the offspring. Manipulating early microbiota through maternal probiotic supplementation has been shown to be an effective means to improve outcomes; however, the mechanisms remain unclear. In this study, we demonstrated that MIA modeled by exposing pregnant dams to lipopolysaccharide (LPS) induced an underdevelopment of the blood vessels, an increase in permeability and astrogliosis of the blood–brain barrier (BBB) at prewean age. The BBB developmental and functional deficits early in life impaired spatial learning later in life. Maternal *Limosilactobacillus reuteri* (*L. reuteri*) supplementation starting at birth rescued the BBB underdevelopment and dysfunction-associated cognitive function. Maternal *L. reuteri*-mediated alterations in β-diversity of the microbial community and metabolic responses in the offspring provide mechanisms and potential targets for promoting BBB integrity and long-term neurodevelopmental outcomes.

## Introduction

Maternal acute and chronic inflammatory states derived from obesity, asthma, autoimmune disorders, depression, pre-eclampsia, and gestational diabetes, as well as environmental risks such as psychosocial stress, low socioeconomic status, exposure to smoking and pollution, and microbial dysbiosis are linked to diverse adverse neurodevelopmental outcomes in children.^1,2^ In particular, maternal inflammation in late-gestation poses increased risk for neurodevelopmental deficits in the offspring.^3–6^ Chorioamnionitis, characterized as bacterial infection resulting in acute inflammation of the placenta and/or fetal membranes, affects up to 10% of pregnancies and is highly associated with adverse neurodevelopmental outcomes, namely, periventricular leukomalacia, neonatal encephalopathy, and cerebral palsy.^7–10^ Rodent models of maternal immune activation (MIA) in response to maternal infection have demonstrated that maternal exposure to lipopolysaccharide (LPS), a bacterial cell wall component of gram-negative bacteria, late in pregnancy (from E17.5 to E20.5) leads to neuronal apoptosis in the cortex, hypomyelination in white matter, neuroinflammation and microgliosis.^11–16^ In addition, these brain developmental changes induced by MIA have long-lasting effects on behaviors including spatial learning and memory, social interaction, anxiety, and motor activity with the degree of impacts depending on the type of the rodents and the dose and timing of the LPS challenge (see detailed review by Bao *et* al^17^). Based on the growing evidence from both clinical and animal studies, approaches to diminish MIA-induced impaired neurodevelopment are needed.

Host development of the brain has been shown to be influenced by early life microbiota development,^18–23^ with accumulating data suggesting communication between the gut microbiota and the central nervous system (CNS) known as the gut-microbiome-brain axis.^18,19,22,24,25^ Thus, optimal gut microbiota development may lead to optimal brain development. Probiotics are defined by the World Health Organization as “live microorganisms, which when administered in adequate amounts, confer a benefit for the host”.^26^ *Limosilactobacillus reuteri* (formerly *Lactobacillus reuteri, L. reuteri*) is a probiotic that exhibits many beneficial traits in gut physiology including production of antimicrobial molecules to prevent pathogen overgrowth and regulation of bacterial colonization, mucosal barrier integrity, mucosal IgA responses and production of anti-inflammatory cytokines.^27,28^ Prophylactic use of *L. reuteri* to treat breastfed infants with colic not only reduces the number and duration of crying episodes^29,30^ but also improves functional gastrointestinal disorders including gastroesophageal reflux and constipation.^31^ In animal studies, a maternal high fat diet induced a dramatic decrease in *L. reuteri* in the microbial community of offspring and *L. reuteri* treatment in the offspring reversed maternal high fat diet-induced social deficits in offspring mice.^32^ However, even though the gut microbiota can be manipulated with probiotics,^33,34^ there are concerns about direct administration of probiotics to newborns due to the immature immune system of newborns, suspected higher susceptibility to infections^35^ and reported sepsis cases when probiotics were given prophylactically to reduce the incidence of necrotizing enterocolitis in preterm infants.^36–38^ Previously, we and others have shown that maternal microbial populations can change behavioral outcomes in the offspring^39^ and that maternal probiotic supplementation after proinflammatory insults can improve brain development in the offspring.^40^ Therefore, optimization of the microbiome and outcomes through maternal probiotic supplementation can be an alternative route to improve neurological outcomes of the offspring.^39^

The mechanisms by which gut microbiota communicate with the CNS are still largely unknown. Suggested potential links include pathways of systemic inflammation, immune surveillance, and production of metabolites/neuromodulators/neurotransmitters.^41^ A common trait of these pathways is the release of microbial mediators (i.e. cytokines, metabolites, activated immune cells) into the systemic system. Whether or what microbial mediators reach the CNS to influence brain function is dependent on the systemic communication between peripheral blood and the tightly regulated CNS barrier known as the blood–brain barrier (BBB). In addition, these microbial mediators might have a direct impact on BBB development and function, and thereby indirectly regulate CNS functions, without even entering the CNS. Dysfunctions of the BBB have been implicated in Parkinson’s and Alzheimer’s disease in adult populations as well as in several neurological disorders in children including cerebral palsy, neonatal stroke, and autism.^42–45^ Pre-clinical studies have suggested that aberrant microbiota and microbiota-related systematic changes can disrupt BBB integrity.^46,47^ However, specific microbial mediators associated with BBB disruption under the influence of maternal inflammation and the resulting influence on offspring neurodevelopment have not been studied.

Some of the properties of the BBB in the developing brain are not yet fully mature at birth, for example, the dominant period of differentiation of astrocytes and the ensheathment of the brain vascular system with astroglial end-feet occurs in rodents in the first 3 weeks of postnatal life.^48^ Given that the BBB is not fully mature at the time of birth, we hypothesized that maternal probiotic administration may partially alleviate the adverse impact of MIA on offspring BBB development and function. We specifically hypothesized that early *L. reuteri* exposure during lactation (days 1–21 of offspring life) would improve MIA-induced BBB dysfunction and neurodevelopment deficits through altering the metabolites that cross the BBB. Herein, we report that MIA, modeled by maternal LPS exposure, induces a development deficit of the vasculature of the BBB. This was associated with distinct shifts in the serum and brain metabolome of the offspring. Maternally administrated *L. reuteri* during lactation normalized MIA-induced BBB development deficits and increased permeability, promoted entry of specific bacterial metabolites to the brain, and significantly improved spatial learning later in life.

## Results

### *Maternal administration of* L. reuteri *during lactation improved spatial learning in the offspring after maternal LPS exposure*

**Figure 1.**
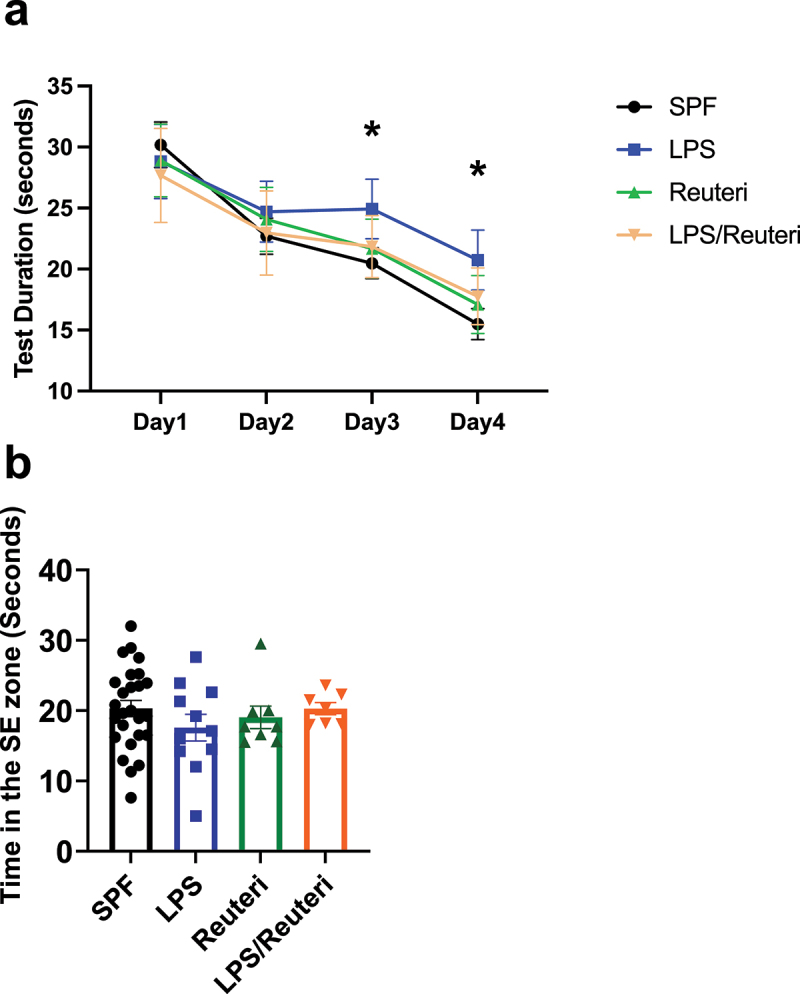
Maternal *L. reuteri* supplementation during lactation rescued spatial learning deficit induced by maternal LPS exposure. (a) Significant difference during training at 12 weeks was found among SPF (n = 26), LPS (n = 11), *L. reuteri* (n = 8) and LPS/*L. reuteri* (n = 7) groups. SPF, *L. reuteri* and LPS/*L. reuteri* mice had significantly higher learning curve slopes than LPS by repeated measurement ANOVA. At training days 3 and 4, LPS mice took significantly more time to locate the escape platform than the mice of the other three treatment groups. Asterisks indicate significant differences of *p*-value at least <0.05. (b) Time in the platform quadrant during the probe trial was not different among the four treatment groups.

Maternal LPS exposure is a widely used animal model of chorioamnionitis and infection associated MIA for the study of long-term outcomes in the offspring.^17^ To determine if the neurodevelopmental outcomes of the offspring from the maternal LPS exposure model could be improved by maternally administrated *L. reuteri* during lactation, Morris water maze was used to assess spatial learning and memory in the offspring at 12 weeks of age. Four testing groups were used SPF (n = 26), LPS (n = 11), Reuteri (n = 8), and LPS/Reuteri (n = 7). In calculating escape latency during testing days by two-way ANOVA with repeated measures (trials), both the main effect factors of testing day (F_3, 192_ = 176.8, *p* < .0001) and treatment (F_3,192_ = 14.68, *p* < .0001) were significant across all groups, demonstrating that the latency time to find the hidden platform decreased during training in all, thus all groups were learning (Figure 1a). However, offspring from the maternal LPS exposure group showed a longer latency to escape onto the hidden platform on the 3rd and 4th day when compared to control, Reuteri or LPS/Reuteri groups (two-way repeated measure ANOVA simple effect by Tukey’s *post hoc* test). Adjusted (for multiple comparison) *p* values at day 3, LPS vs SPF *p* < .0001, LPS vs Reuteri *p* = .0089, LPS vs LPS/Reuteri *p* = .0203; at day 4, LPS vs SPF *p* < .0001, LPS vs Reuteri *p* = .0024, LPS vs LPS/Reuteri *p* = .0279). These data demonstrate that maternal LPS exposure impaired spatial learning ability when compared to controls, but that *L. reuteri* supplementation during lactation reversed the spatial learning impairment. In the probe trial, time spent in the quadrant where the hidden platform had been (Figure 1b) did not differ among the four groups, indicating that spatial memory was not affected.

### *Maternal* L. reuteri *supplementation during lactation restored maternal LPS-induced impairment of brain vascular development and BBB hyperpermeability in the offspring*

To determine if the alteration in the offspring spatial learning associated with maternal LPS exposure was linked to early BBB dysfunction, we first evaluated the overall brain vascular development. Since studies have shown that rodents are only susceptible to inflammation-induced increased permeability of cerebral blood vessels before P20,^49^ a stage of brain development equivalent to 22–40 weeks of gestation in humans,^50^ in the current study mice at 2-week old were subjected to MRI time of flight (TOF) to visualize flow within vessels, without the need to administer contrast.

**Figure 2.**
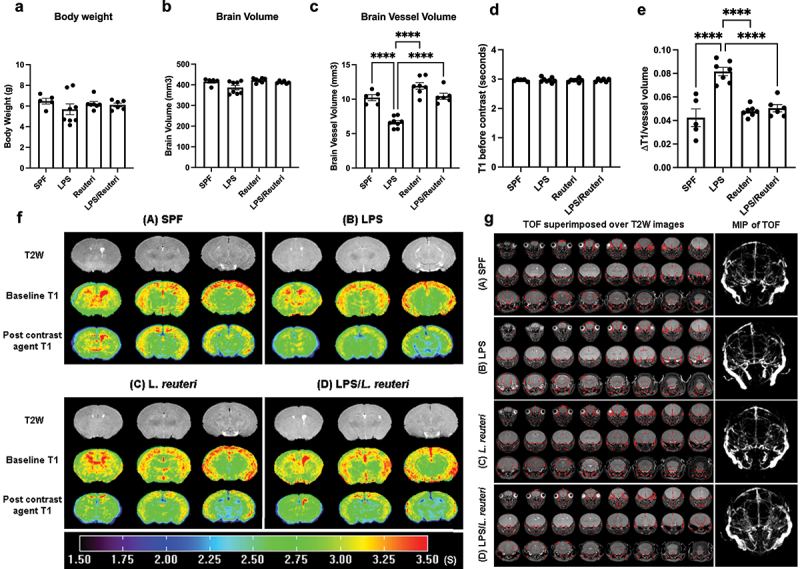
*L. reuteri* supplementation starting at birth reversed gestational LPS-induced vascular development deficits and hyperpermeability of the BBB in the offspring. Treatment did not affect (a) body weights or (b) total brain volume. (c) Maternal LPS significantly decreased the brain vascular volume compared to the saline control group and *L. reuteri* supplementation during lactation significantly minimized the LPS-induced vascular volume deficit (n = 5,8,7,6, respectively). Bars with ⊓ denote significant difference between experimental groups (****p < .0001, one-way ANOVA). (d) Baseline T1 values (seconds) were not different among the treatment groups. (e) Maternal LPS significantly increased the BBB permeability compared to the saline control offspring group and *L. reuteri* supplementation during lactation significantly minimized the LPS-induced BBB hyperpermeability. Quantification of permeability was derived from baseline T1 and post contrast (gd) T1 values. Permeability is presented as ΔT1 (baseline T1-post contrast T1)/vessel volume. Bars with ⊓ denote significant difference between experimental groups (****p < .0001, one-way ANOVA). (f) Representative T2W and T1 images of brains. Panels represent treatment groups (A) SPF, (B) LPS, (C) *L. reuteri*, and (D) LPS/*L. reuteri*. 1st row (gray) -Three middle slices of T2W brain images; 2^nd^ row – measured mouse brain T1 maps before contrast agent injection; 3^rd^ row – measured mouse brain T1 maps 25 minutes after contrast agent injection. The color bar underneath the maps shows scales (value) of the T1 map. (g) Representative images of mouse brain blood vessels (red color) obtained from the TOF datasets superimposed over T2W images (gray). Panels represent treatment groups (A) SPF, (B) LPS, (C) *L. reuteri*, and (D) LPS/*L. reuteri*. For visual inspection, Maximum Intensity Projection (MIP) image shown in the right column was generated from TOF datasets. The MIP connects the high intensity dots of the blood vessels in three dimensions.

Mouse body weights at 2 weeks of age were not different (Figure 2a, *p* > .05, ANOVA) among the treatment groups. There was also no statistical difference in brain volume among the treatment groups (quantification in Figure 2b, *p* > .05, ANOVA) based on the T2w imaging of brain anatomy (Figure 2f top panel).

However, prenatal exposure to LPS compared to saline SPF control group did significantly decrease the total blood vessel volume based on TOF measurement (Figure 2g (A) and 2 g (B), quantification in Figure 2c, *p* < .0001, Tukey’s *post hoc* test after one-way ANOVA). *L. reuteri* supplementation alone during lactation did not affect the vascular volume (Figure 2g (C)) but reestablished the vascular volume when compared to the maternal LPS challenged alone group (Figure 2g (D), p < .0001, Tukey’s *post hoc* test after one-way ANOVA).

BBB permeability was evaluated by calculating ΔT1 (longitudinal relaxation time) value between the before contrast T1 value (baseline T1 in Figure 2f middle panel) and post-contrast agent T1 value (Figure 2f bottom panel). Although there was no statistical difference between the average baseline T1 values (Figure 2d), the amount of contrast in the brain was significantly higher in the maternal LPS challenged group when compared to the control group (Figure 2e, p < .0001, Tukey’s *post hoc* test after one-way ANOVA), indicating higher BBB permeability in the maternal LPS exposed offspring at 2 weeks of age. Remarkably, maternal *L. reuteri* administration during lactation repaired the prenatal LPS-induced BBB hyperpermeability (Figure 2e, p<.0001, Tukey’s *post hoc* test after one-way ANOVA). These data demonstrate for the first time that gestational MIA-induced by LPS significantly impairs vascular development and permeability of the BBB in the offspring and that *L. reuteri* supplementation starting at birth can reverse these deficits.

### *Maternal administration of* L. reuteri *during lactation ameliorated astrogliosis in the offspring after maternal LPS exposure*

**Figure 3.**
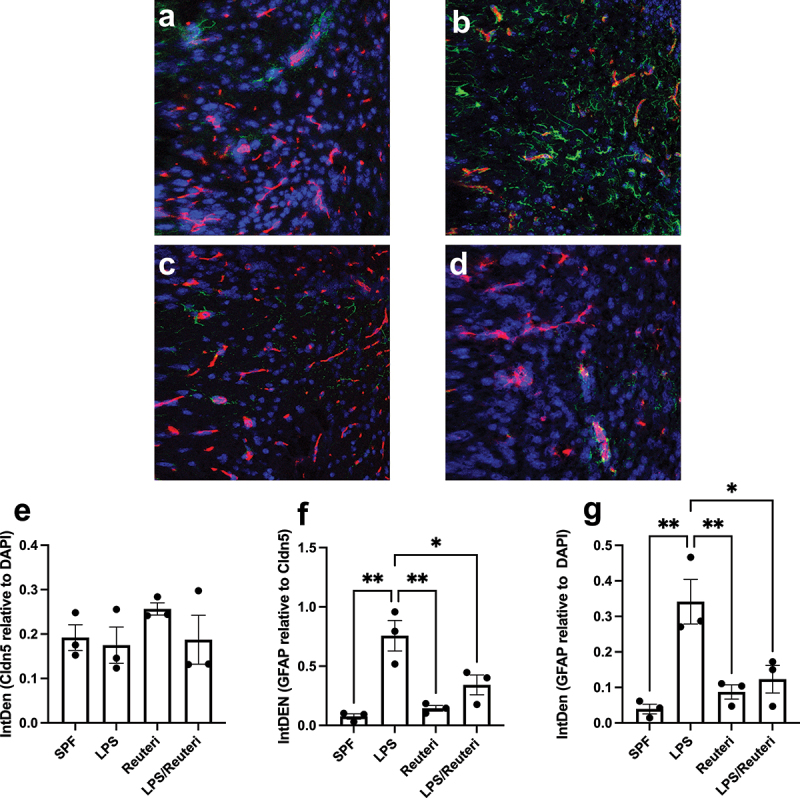
Astrogliosis in 2-week-old offspring induced by maternal LPS was reduced by maternal *L. reuteri* supplementation. Representative images of fluorescence microscopy of claudin-5 (location of the brain capillaries, red), GFAP astrocyte (green), and DAPI (nuclei, blue). Seven to ten sections per mouse of three mice were inspected in each group. Stronger than control SPF GFAP staining (a) was observed around the blood vessel and in the brain with maternal LPS insult (b). Maternal supplemented of *L. reuteri* (c) without or (d) with maternal LPS had GFAP levels similar to the control group. Based on quantification of astrocyte activation using ImageJ (NIH), (e) Overall expression of claudin-5 was not affected by treatment. (f) GFAP expression in the vicinity of the blood vessel and (g) GFAP expression in the brain were expressed as GFAP integral density (IntDen) levels over claudin-5 levels. Bars with ⊓ denote significant difference between experimental groups (all n = 3, at least *p* < .05).

Astrogliosis is a common feature of astrocytes during BBB disruption, characterized by upregulation of the phenotypical astrocyte protein glial fibrillary acidic protein (GFAP) during CNS insults.^51^ To determine if maternal LPS-induced changes in BBB were linked to astrocyte activation, we performed immunohistochemical staining for GFAP (Figure 3, green) and the BBB-specific tight junction protein claudin-5 (Figure 3, red) to define brain blood vessels. We did not find any differences in claudin-5 protein levels (Figure 3a, SPF; Figure 3b, LPS; Figure 3c, Reuteri; Figure 3d, LPS/Reuteri) quantified by integrated intensity (IntDen) levels (using ImageJ (NIH)) in the cerebrum of the offspring from either maternal *L. reuteri*-supplemented or un-supplemented groups with or without prenatal LPS insult (Figure 3e, p>.05, ANOVA). Maternal LPS challenge did induce a significant elevation in GFAP staining (green) when compared to saline (Figure 3a), both around the blood vessel (identified by claudin-5 staining, red) and in the cerebral tissue (Figure 3b), with quantification presented as GFAP IntDen over claudin-5 IntDen in Figure 3f (*p* = .0012) and GFAP over DAPI IntDen (nuclei staining) in Figure 3g (*p* = .0088), respectively (Tukey’s *post hoc* test after one-way ANOVA). Maternal *L. reuteri* exposure during lactation did not change the GFAP expression but significantly diminished maternal LPS-induced increased GFAP expression (Figure 3c,d, with quantification in Figure 3f *p* = .0247, and 3 g *p* = .0381, respectively, Tukey’s *post hoc* test after one-way ANOVA). These data demonstrate that BBB susceptibility to maternal-LPS-induced disruption is complemented by astrogliosis and that maternal *L. reuteri* during lactation can reduce astrogliosis at both a global brain level and specifically at the BBB.

### *Maternal administration of* L. reuteri *during lactation altered offspring microbiome β-diversity after maternal LPS exposure*

**Figure 4.**
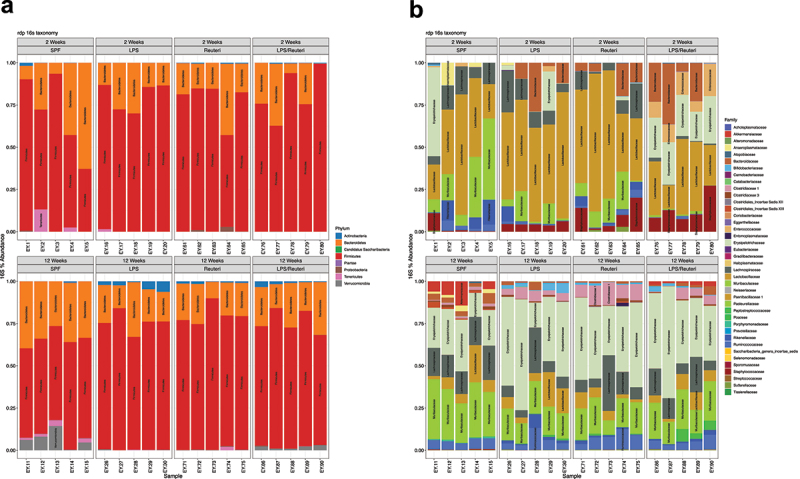
Relative abundance of bacterial communities among the treatment groups. (a) Relative abundance at phylum level of 2 (top) and 12 (bottom) weeks old fecal samples. (b) Relative abundance at family level of 2 (top) and 12 (bottom) weeks old fecal samples (all n = 5).

**Figure 5.**
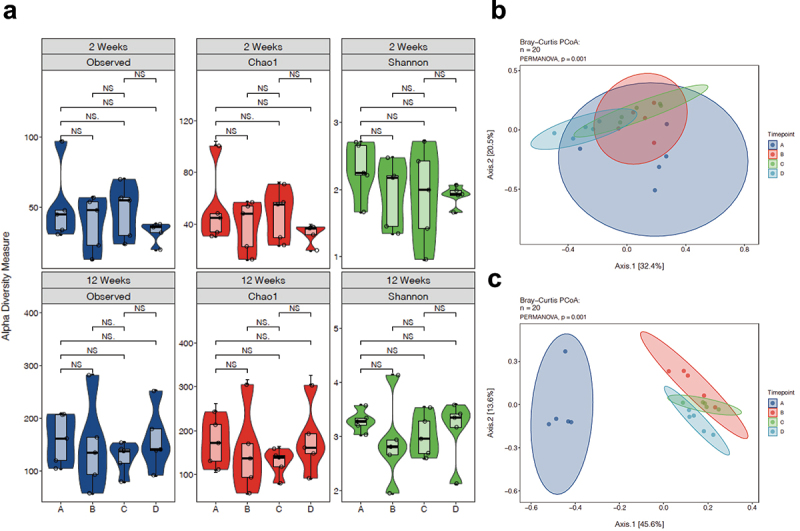
α-diversity and Bray-Curtis principal component analysis of fecal microbiota. α-diversity metrics of (a) observed, chao1, and Shannon diversity of 2- and 12-week-old mouse fecal samples calculated using R package. No significant difference was found among the treatment groups in any of the metrics. Principal component analysis (PCoA) scores are plotted based on the relative abundance of fecal microbiota at the genus level of (b) 2- and (c) 12-week-old mouse fecal samples. The percentage of variation explained by the principal component is indicated on the axis. A. SPF B. LPS C. Reuteri D. LPS/Reuteri. Significant separation in the gut microbiome composition (β-diversity) was observed among different treatment groups (all n = 5) by PERMANOVA (*p* = .001).

Since microbial community composition based on β-diversity has been associated with cognitive functions in other studies,^52^ we investigated the impact of *L. reuteri* exposure on the offspring microbiome. Gut microbiome analysis based on relative abundance demonstrated no significant differences in taxa at the phylum or family levels among the four treatment groups (Figure 4a,b, all groups n = 5). There was also no difference in α-diversity indices at 2 and 12 (Figure 5a) weeks of age with either maternal LPS or *L. reuteri* supplementation during lactation. In contrast, the β-diversity of the gut microbiome among different groups showed differences as reflected in the principal coordinate analysis plot (PCoA) with Bray-Curtis dissimilarity at both 2 (Figure 5b) and 12 weeks of age (Figure 5c). PERMANOVA analysis revealed that there was a distinction in β diversity among treatment groups at both ages (both *p* = .001). Our data demonstrate that maternal LPS exposure and/or *L. reuteri* supplementation during lactation modulates the β diversity of the gut microbiome.

### *Maternal administration of* L. reuteri *during lactation reshaped metabolomic profile shifts induced by maternal LPS exposure*

To test whether the bacterial composition changes observed in our study based on β-diversity are associated with altered metabolic features, we subjected both serum and brain samples to metabolite profiling. This non-targeted metabolomic experiment resulted in 11,054 features. To reduce the information burden for interpretation of the data, features were computationally assigned putative molecular IDs using the GNPS online platform and only features that could be assigned an analogous match to fragmentation spectra found in the library were included in the analysis. Future feature list filtering as described in the Methods resulted in 389 high-quality features with putative IDs. To determine if overall metabolic profiles were affected by the different treatment groups, a principal component analysis was used to perform unsupervised clustering based on feature abundances.

**Figure 6.**
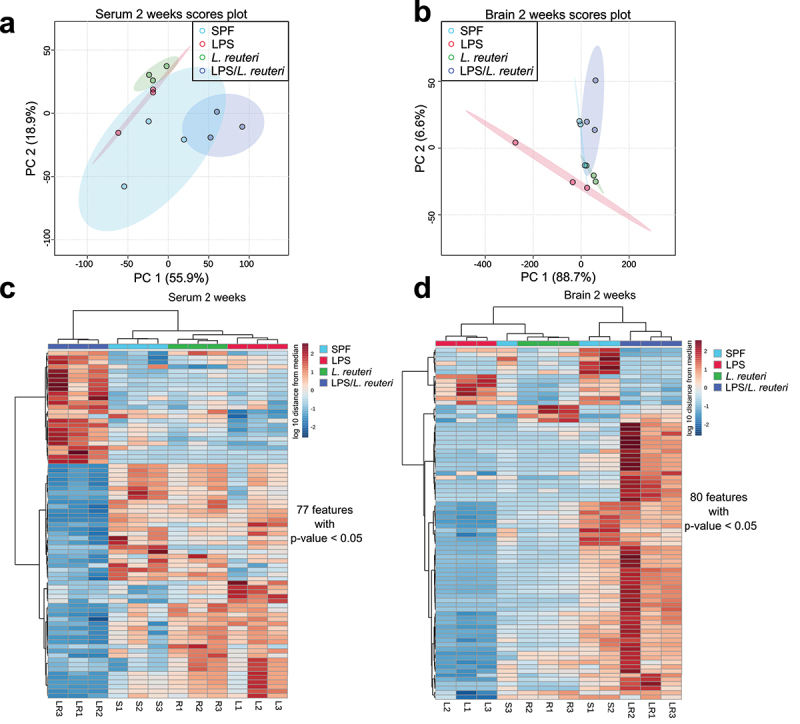
Principal component analysis and heatmap of serum and brain metabolite profiles at 2 weeks of age. Principal component analysis (PCoA) scores are plotted based on the normalized peak area of (a) serum and (b) brain metabolites of 2-week-old mice. A Hierarchical clustering was applied to arrange the metabolites based on the similarity of the abundance among samples. For 2-week-old samples, (c) 77 significantly different serum features and (d) 88 significantly different brain features were plotted (One-way ANOVA test with Benjamini–Hochberg method-adjusted p value <.05, all n = 3).

**Figure 7.**
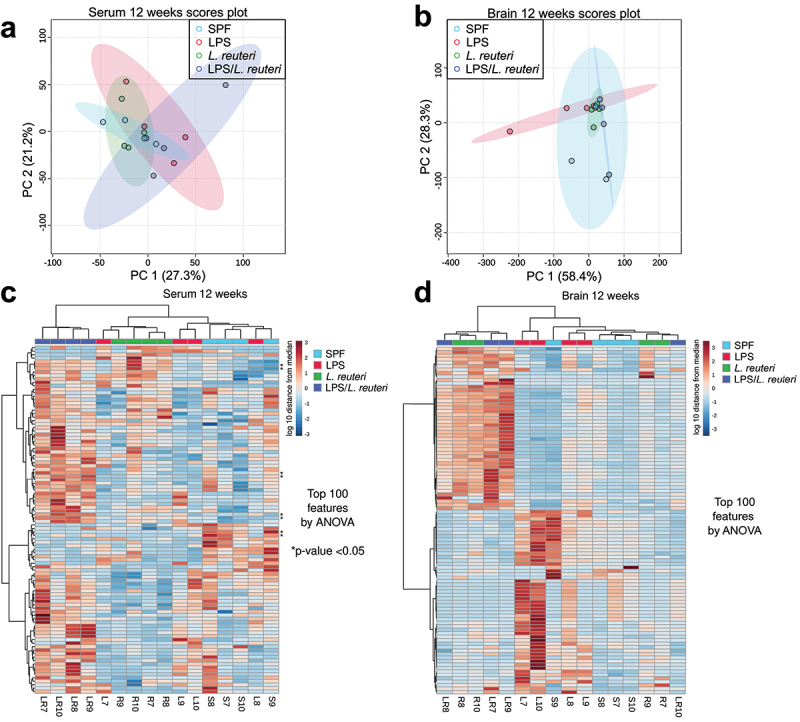
Principal component analysis and heatmap of serum and brain metabolite profiles at 12 weeks of age. Principal component analysis (PCoA) scores are plotted based on the normalized peak area of (a) serum and (b) brain metabolites of 12-week-old mice. For 12-week-old samples, top 100 features by ANOVA were plotted for (c) serum and (d) brain samples with * indicating a significant difference among the four treatment groups (One-way ANOVA test with Benjamini–Hochberg method-adjusted *p* value <.05, all n = 4).

At 2-week old, metabolic profiles in both serum (Figure 6a, all n = 3) and brain (Figure 6b, all n = 3) samples clustered separately among the four treatment groups, with the clearest distinction in brain metabolic profiles observed in the LPS group compared to the other three groups. The distinct compositional clustering by treatments at 2 weeks of age is further demonstrated in the heatmaps of significantly differential abundance of the features shown in Figure 6c (serum with 77 features annotated) and Figure 6d (brain with 80 features annotated) based on one-way ANOVA with FDR (cutoff at 0.05)-adjusted *p* value (see Table S1 for the complete list of metabolites and Table S2 for ANOVA table). At 12 weeks of age, there was no separation in either the serum (Figure 7a, all n = 4) nor the brain (Figure 7b, all n = 4) metabolic pools. Using the same approach, at 12 weeks of age, the abundance of eight features in the serum (Figure 7c) (see Table S2 for the list of metabolites) and no feature in the brain (Figure 7d) were significantly different among the treatment groups as shown in the heatmaps.

**Figure 8.**
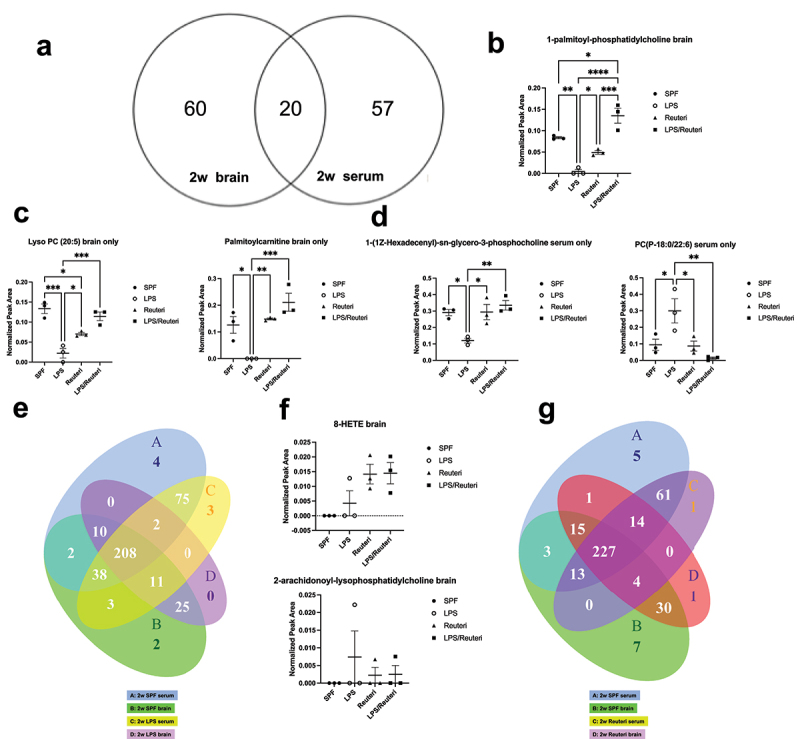
Significantly different metabolic features between the serum and brain pool. Features were presented using their putatively identified names. (a) Venn diagram showing the significantly different metabolic features between the serum and brain pool. The number at the intersection represents the number of significantly different metabolites shared by serum and brain, while the number out of the intersection represents the number of unique metabolites in each pool. (b) Of the shared metabolites in both pools, 1-palmitoyl-phosphatidylcholine in the brain of the LPS group was significantly less than that of the SPF group (*p* = .0016). 1-palmitoyl-phosphatidylcholine levels in both Reuteri and LPS/Reuteri groups were significantly higher than that of the LPS group (*p* = .048 and *p* < .0001, respectively). (c) In the unique brain pool, both Lyso PC (20:5) and palmitoylcarnitine in the brain of LPS group were significantly less than that of the SPF group (*p* = .0003 and *p* = .0197, respectively). These two metabolite levels in both Reuteri and LPS/Reuteri groups were significantly higher than that of the LPS groups (for Lyso PC (20:5), *p* = .045 and *p* = .0009, respectively; for palmitoylcarnitine, *p* = .0079 and *p* = .0009, respectively). (d) In the unique serum pool, 1-(1Z-Hexadecenyl)-sn-glycero-3-phosphocholine level in the LPS group was significantly less (*p* = .016) and PC(P-18:0/22:6) (*p* = .039) level was significantly higher than that of the SPF group. These two metabolite levels in both the Reuteri and LPS/Reuteri groups were similar to that of the SPF group. All data were analyzed by one-way ANOVA with Tukey’s post hoc test, all n = 3. Unique features using their putatively identified names that crossed the BBB of the offspring upon maternal LPS challenge were identified in (e) Venn diagram, revealing that there were two features that uniquely crossed the BBB under the influence of LPS. (f) The levels of 8-HETE and (c) 2-arachidonoyl-lysophosphatidylcholine were not different among the four treatment groups (one-way ANOVA). Unique features that crossed the BBB of the offspring upon maternal *L. reuteri* exposure during lactation were identified in (g) Venn diagram, revealing that there were 14 unique features that crossed the BBB under the influence of *L. reuteri.*

Out of the 77 serum and 80 brain significantly differential features from 2-week-old mice, 20 were present in both pools, 60 were only in the brain pool and 57 features were only in the serum pool (Figure 8a, see full list from Table S2). We next specifically identified the features associated with LPS that were altered by *L. reuteri*. Of the 20 features present in both pools, the levels of a lysophosphatidylcholine feature putatively identified as 1-palmitoyl-phosphatidylcholine (LysoPC(16:0)) were significantly decreased in the brains of LPS treatment group (*p* = .0016) and restored by *L. reuteri* exposure (Figure 8b, p<.0001). Of the 60 features only present in the brain, the levels of two features putatively identified as LysoPC (20:5) and palmitoylcarnitine levels were reduced in the offspring brains of the maternal LPS-treated group but reestablished by *L. reuteri* exposure during lactation to levels similar to the SPF controls (Figure 8c, for LysoPC (20:5), SPF vs LPS, *p* = .0003, LPS vs LPS/Reuteri, *p* = .0009; for palmitoylcarnitine, SPF vs LPS, *p* = .0197, LPS vs LPS/Reuteri, *p* = .0009). In the serum only pool, *L. reuteri* during lactation repaired maternal LPS-induced lower levels of a phosphatidylcholine feature annotated as of 1-(1Z-Hexadecenyl)-sn-glycero-3-phosphocholine (LPS vs LPS/Reuteri, *p* = .0042) and reduced the maternal LPS-induced higher levels of a phosphatidylcholine feature putatively identified as PC(P-18:0/22:6) (Figure 8d, LPS vs LPS/Reuteri, *p* = .0066). Of the eight features identified with overall significant different abundance in the 12-week serum pool, no features associated with LPS were altered by *L. reuteri*.

To determine whether maternal LPS exposure induces new metabolic features that cross the BBB, we used a Venn diagram to sort features that were in the serum only pool in the untreated SPF but that appeared specifically in the brain of LPS-treated group (we excluded those also present in the LPS serum pool to focus investigation on those that newly crossed the BBB in response to LPS, Figure 8e). An eicosanoid putatively identified as 8-hydroxyeicosatetraenoic acid (8-HETE) and a lysophosphatidylcholine feature putatively identified as 2-arachidonoyl-lysophosphatidylcholine, both derivatives of arachidonic acid, met these criteria. The relative abundance of these features was not significantly different among the treatment groups in the serum alone (data not shown) or brain alone pools of metabolites (Figure 8f).

*L. reuteri* supplementation alone during lactation resulted in 14 new metabolites that crossed the BBB to the brain when compared to the untreated SPF controls (Figure 8g and see Table 1 with the list of features). The majority of these metabolites with assigned putative IDs were lipid and lipid-like molecules that were bile acid or alcohol derivatives. Interestingly, none of these 14 metabolites were present in the SPF or LPS brain pools. Inspection of raw data revealed that these 14 features fell below the chosen noise and peak shape cutoffs for the curated data set in the brains of SPF and LPS mice suggesting that they were increased in the brains of *L. reuteri* exposed mice as a result of *L. reuteri*-associated metabolism.

## Discussion

Despite mounting evidence suggesting that the gut microbiome plays a role in regulating brain function, the exact mechanisms remain unclear. This study demonstrates that gestational MIA disrupts vascular formation and permeability early in life (before weaning), promotes astrocyte overactivation and results in behavioral alterations in spatial learning later in life. *L. reuteri*, an indigenous member of the human gut microbiome, when administered to lactating dams, was shown to be an effective mean of rescuing the developmental deficits in the BBB and long-term cognitive function in their offspring. These findings were furthered supported by early changes (at 2-week old) in both the serum and brain metabolite profiles induced by gestational MIA and *L. reuteri* exposure during lactation. Specifically, metabolic products of lipid/bile acid metabolism associated with *L. reuteri* exposure during lactation can cross the BBB and may represent targets with potential beneficial bioactivities to remediate MIA insults. Notably, metabolic profiles at 12-week old, the time of observed differential cognitive function, were largely not affected by MIA or *L. reuteri* early life exposure. These findings suggest that there is an early developmental window key to optimization of the BBB through probiotics, and that this early optimization has long-term impact on neurological outcomes later in life. In addition, our approach to administer *L. reuteri* to the mother during lactation indicates that the beneficial effects of probiotics can be attained without directly exposing newborns to the probiotics. This is of clinical relevance considering that probiotics could be a potential therapeutic agent to improve offspring outcomes when adverse conditions associated with MIA or chorioamnionitis are diagnosed. Together, our findings point to early BBB vasculature development as important for long-term cognitive function and as an early biomarker of gut-brain axis function.

Strong evidence has suggested that MIA contributes to cognitive deficits in offspring in both rodents and humans.^49^ Mechanisms by which MIA can mediate brain development and subsequent cognitive outcomes were proposed to be via both systemic and local inflammation.^6,53^ For example, LPS-simulated inflammation during pregnancy induced brain injury through activation of neuronal nitric oxide synthase (NOS) and NF-κB pathways in the offspring brains.^6^ Previous studies also suggested that gestational MIA disruption of BBB could be an etiological contributor to neuropsychiatric disorders.^46,54^ In this study, we demonstrate that MIA (LPS) is a potent inducer of altered BBB development and function and CNS dysfunction. At 2 weeks of life rodent age, a stage of brain development equivalent to 22–37 weeks of gestation in humans,^50^ we observed increased permeability of the BBB and the novel finding of a significant BBB developmental deficit shown by the reduced total blood vessel volume.

It has previously been shown that probiotics can modulate BBB integrity.^47^ Germ-free mice colonized with a commensal microbiota, or a short-chain fatty acid-producing bacterial strain, reduced BBB permeability and normalized the expression of tight junction proteins.^47^ We have also previously published that maternal probiotic supplementation can improve offspring’s BBB functions upon inflammatory insult.^40^ In our current study, pregnant SPF mice were given *L. reuteri* daily from birth to weaning (P21). Given that the process of the ensheathment of endothelial cells with astroglial end-feet occurs postnatally and BBB disruption is highly associated with reactive gliosis, our data demonstrate that maternal *L. reuteri* supplementation immediately after birth can specifically target astrocytes at both BBB and global levels in addition to normalizing MIA-induced developmental deficits in vessel development and increased BBB permeability.

To investigate the mechanism by which an altered microbiome may impact the brain, we studied the metabolic responses to MIA and *L. reuteri* on both vascular and brain sides of the BBB. We identified an eicosanoid annotated as 8-hydroxyeicosatetraenoic acid (8-HETE) and a lysophosphatidylcholine annotated as 2-arachidonoyl-lysophosphatidylcholine as able to cross the BBB of the offspring after maternal LPS challenge. Out of the 20 differentially present metabolites in both serum and brain pools, decreased level of a lysophosphatidylcholine annotated as Lyso PC (16:0) in the brain associated with maternal LPS was restored by *L. reuteri* exposure. The importance of microbiome associated metabolic features on the brain may not just be what crosses to the brain, but also what impacts BBB development directly. In the serum, where potentially metabolites would have direct impact on BBB from the vascular side, *L. reuteri* modulated maternal LPS-induced decreased levels of a lysophosphatidylcholine annotated as1-Hexadecyl-sn-glycero-3-phosphocholine and increased levels of another lysophosphatidylcholine annotated as PC(P-18:0/22:6) to levels similar to the controls. Lysophosphatidylcholine has been shown *in vitro* to induce endothelial cell injury through oxidative stress,^55^ however, whether these two metabolites have any effects on the endothelial cells of the BBB has yet to be determined.

*L. reuteri* exposure itself resulted in 14 additional metabolites that crossed the BBB. These metabolites are mostly related to lipid/bile acid metabolism. Bile acids regulate host glucose and lipid metabolism and absorption of fat and fat-soluble vitamins.^56^ Bile acids and metabolic products of bile acids have also been implicated in the communication between the gut microbiota and brain.^57^ Both primary bile acid and conjugated bile acids can cross the BBB but with different mechanisms. Primary bile acids cross the BBB through diffusion while conjugated bile acids utilize yet unidentified transporters on the BBB.^58^ The molecular role of bile acids in the CNS is largely unknown,^59^ however, different bile acid byproducts can have different effects on BBB function. The primary bile acids chenodeoxycholic acid and deoxycholic acid in the circulation can disrupt tight junctions and increase permeability of the BBB.^60^ Ursodeoxycholic acid (UDCA) and its glycine-conjugated form GUDCA protect brain endothelial cells from apoptosis.^61^ Rodent taurine-conjugated UDCA reduces neuroinflammation through its brain TGR5 receptor^62^ and has been implicated in several neurodegenerative diseases.^58^ Therefore, we propose that the protective effects on the BBB development and improved spatial learning by *L. reuteri* might be due to *L. reuteri*-induced bile acid-related metabolism.

Other studies have shown that mice consuming *L. reuteri* or a sterile *L. reuteri* lysate can increase the plasma level of oxytocin, a neuromodulator of social behavior, stress regulation, and learning and memory.^32,63,64^ The exact mechanism of which *L. reuteri* can upregulate oxytocin production is not clear; however, the fact that *L. reuteri* lysate had the similar effect as the live probiotics suggesting that a bacterial component, potentially to-be-determined metabolites, can be the key regulators. Oxytocin can cross BBB by the receptor for advanced glycation end-products (RAGE) in the endothelial cells resided at the BBB,^65^ and it would be interesting to investigate in future experiments whether RAGE is involved in the effects of MIA and *L. reuteri* we observed in this study.

Limitation of this study includes the low number of samples for metabolomics in each treatment group (at 2 weeks of age all n = 3 and at 12 weeks of age all n = 4). The intention of this study was to shift the paradigm of the current gut microbiome-brain axis study. Our study suggests that the communication between gut microbiome and the brain might rely on the effects of systemic microbial metabolites on BBB development itself in addition or on the brain if they can reach the brain through BBB.

We acknowledge that there is a well-documented sexual dimorphism in behaviors in clinical and animal studies. Our main hypothesis is that MIA can induce BBB development deficit and *L. reuteri* can rescue BBB development, and we did not hypothesize that sex would be a factor to influence BBB susceptibility by MIA in our study. Furthermore, C57BL/6 J might not be a good strain to detect sex difference in behaviors. According to the literature, most studies using C57BL/6 mice do not exhibit sex dimorphism in spatial task performance in the Morris water maze test.^66–70^ C57BL/6 J female mice have been previously reported to be more anxious in elevated plus maze test than the male counterparts but no difference was detected between the sexes in exploratory activities in the open field test.^71^ There has also been no sociability and novelty difference between the female and male C57BL/6 J mice in the three-chamber test.^72^ Our previous publication^23^ also agrees with most of the studies in C57BL/6 J mice where we did not find significant differences between the sexes in open field, elevated plus maze, fear conditioning test, three-chamber social test, and Morris water maze test. Therefore, both female and male animals were used in this study. Maternal *L. reuteri* (from late gestation to wean) has recently been shown to shape the microbiome of offspring in a sex-dependent manner.^73^ Maternal *L. reuteri* induced a progressively increasing microbiome separation in the pups from controls in both female and male mice based on β diversity. However, the relative abundance of *Lactobacillus, Akkermansia, Lachnoclostridium*, and *Bacteroides* following maternal *L. reuteri* supplementation at P70 was significantly increased in female mice but not in male mice. Since we did not hypothesize that MIA would affect BBB development in a sex-dependent manner, future studies evaluating the effects of maternal *L. reuteri* on MIA-induced BBB deficits in both sexes are warranted.

In conclusion, MIA induced a developmental deficit in offspring vasculature formation associated with disrupted BBB integrity. BBB development deficiency early in life was associated with long-lasting effects on cognitive function. By introducing *L. reuteri* as an early microbial intervention during lactation, we were able to improve BBB development and cognitive function. Regulation of metabolic responses to MIA through *L. reuteri* at both the vascular and brain sides of the BBB provides a potential mechanism and targets for promoting BBB integrity and long-term neurological outcomes.

## Methods

### Animals

Animal care and experimental procedures were approved by the University of Chicago Institutional Animal Care and Use Committee strictly in accordance with all guidelines by the U.S. National Institutes of Health. Timed pregnant C57BL/6 J mice were kept on a 12-hour light/dark cycle and had access to food and water *ad libitum*. At gestational day 16 (E16), dams were randomized for injections of equivalent volumes (200 µL) of intraperitoneal (i.p.) LPS from Escherichia coli O55:B5 (Sigma-Aldrich, St. Louis, MO, USA) (50ug/kg body weight of dam) or saline. *Lactobacillus reuteri* (ATCC PTA 6475) (*L. reuteri*) was cultured in an anaerobic cabinet (10% CO_2_, 5% H_2_, and 85% N_2_) overnight at 37°C in MRS broth. Bacteria were spun down and suspended in an equivalent volume of serum-free DMEM medium. Right after delivery, both vehicle and LPS-challenged dams were further randomized to be fed daily (orally gavaged in a volume of 100 µL) with 10^9^ *L. reuteri* or vehicle until weaning or time points before weaning when pups were sacrificed for tissue collection or transferred to MRI. Another subset of pups was allowed to grow to the age of 12 weeks and subjected to behavioral testing. This resulted in four study groups: control, LPS, *L. reuteri*, LPS/L. *reuteri*.

### Morris water maze

Morris water maze was used to evaluate spatial learning and memory as previously described.^23^ Animal movements were registered and processed with ANY-maze software (Stoelting Co., Wood Dale, IL). Animal number in each treatment group is SPF (n = 26, 13 females and 13 males from six litters), LPS (n = 11, three females and eight males from three litters), Reuteri (n = 8, two females and six males from three litters), and LPS/Reuteri (n = 7, three females and four males from two litters). Briefly, mice were placed in a circular 120 cm diameter tank with room temperature (22°C) water. At the training stage of the test, the mice were trained to locate a visible 10 cm diameter platform exposed 1 cm above the water. Five trials were performed, and the platform location was changed for each trial. At the testing stage, mice were allowed to find the hidden platform that was submerged 1 cm below the surface in the southeast quadrant. Mice were tested for four consecutive days. On all testing days, each mouse was subjected to five trials, each with a different starting position. The latency required to locate the platform (test duration, no more than 60 s) was recorded. The probe trial was performed on the 5th day, in which mice swam for 60 seconds with no platform in the tank. Time spent in the quadrant where the submerged platform had been in previous stages was recorded.

## MRI experiments

### Protocol

Animals were anesthetized prior to imaging experiments, and anesthesia was maintained during imaging with 1.5–2.5% isoflurane. Temperature, heart and respiration rates were monitored and kept within normal range with a fiber optic detection system from SA Instruments (Stony Brook, NY, USA), designed for use in small animals. Animal number in each treatment group is SPF (n = 5, two females and three males from two litters), LPS (n = 8, five females and three males from four litters), Reuteri (n = 7, four females and three males from three litters), and LPS/Reuteri (n = 6, two females and four males from two litters).

MRI data were acquired on a 9.4 Tesla small animal scanner (Bruker, Ettlingen, Germany) with 11.6 cm inner diameter actively shielded gradient coils (maximum constant gradient strength for all axes – 230 mT/m). Each mouse was placed supine on an animal holder and inserted into a 30 mm diameter quadrature volume coil (Rapid MR International, Columbus, OH). To cover the whole brain, multi-slice spin echo T_2_-weighted (T_2_W) imaging was acquired along the coronal direction with a RARE (Rapid Acquisition with Relaxation Enhancement) pulse sequence (repetition time (TR) = 4000 ms, echo time (TE)_effective_ = 24 ms, field-of-view (FOV) = 25.6 × 19.2 mm^2^, matrix size = 256 × 192, slice thickness = 0.5 mm, RARE factor = 8, number of excitations (NEX) = 4). For the same geometry as T2W imaging, time-of-flight (TOF) angiographic images were acquired with a flow compensated T_1_-weighted sequence (TR/TE = 15/3.9 ms, flip angle = 60°, FOV = 25.6 × 19.2 mm^2^, 256 × 192, slice thickness = 0.5 mm). Native T_1_ measurement was performed using RARE VTR (variable TR) images (TR = 281, 350, 500, 1000, 1500, 2000, 3000, 5000, 10000 ms, TE = 12.3 ms, RARE factor = 4, FOV = 25.6 × 19.2 mm^2^, matrix size = 128 × 96, thickness = 1.5 mm, number of slice = 9, NEX = 1). Fifteen minutes after IP injection of 0.1 mmol/kg of Omniscan (gadodiamide, GE Healthcare, USA), the same T1 measurement as above was repeated twice.

### Data analysis

MRI data were analyzed using IDL 6.4 (Harris Geospatial Solutions, Inc. CO, USA) with an in-house software package. The brain region-of-interest (ROI) was manually traced onto the T2W image and superimposed on the TOF imaging and T1 measurement imaging. The whole brain volume was calculated by the sum of all pixels in each slice and multiplied by the slice thickness. To determine the volumes of blood vessels inside the brain, thresholds were set to select only for those pixels representing blood vessels. Using these pixels, the total volumes of blood vessels were calculated.

### Calculation of T_1_

T_1_ maps before and after contrast agent injection were calculated by fitting RARE VTR signal intensity (S_TR_) in each pixel as a function of TR as follows:

$$S_{TR}=P_{0}\cdot \left(1-e^{-TR/T_{1}}\right)$$

where P_0_ is the equilibrium signal depending on the proton density.

## Immunohistochemistry

Brains were freshly obtained from mice at postnatal age of 2 weeks and embedded and frozen in OCT. Eight μm sections were fixed in ice-cold methanol for 20 minutes at −20°C. The samples were permeabilized with PBST with for 15 mins and then incubated with blocking solution (5% goat serum) in 0.2% Triton-X PBS (PBST) for 1 hour at room temperature (RT). The brain sections were then incubated with respective 50 μL of primary antibody solution overnight at 4°C. After wash with PBST four times for 10 mins, the sections were incubated with respective fluorophore-conjugated secondary antibodies for 1 hour at RT. DAPI-antifade mounting medium was used to counterstain nuclei (Invitrogen Inc., Carlsbad, CA, USA). Images were captured with a Stellaris confocal microscope (Leica Microsystems, Inc., Buffalo Grove, IL, USA). ImageJ (U. S. National Institutes of Health, Bethesda, Maryland, USA, http://imagej.nih.gov/ij/, 1997–2012)113 was used for imaging processing and analysis.

## 16S rRNA sequencing

Mouse fecal samples were submitted to the Microbiome Metagenomics Facility of the Duchossois Family Institute (DFI) at the University of Chicago (Chicago, IL, USA) for genomic DNA extraction and subsequent 16S rRNA gene sequencing on the Illumina MiSeq platform. Dada2 (v1.18.0) as our default pipeline was used for processing MiSeq 16S rRNA reads with minor modifications in R (v4.0.3). Specifically, reads were first trimmed at 210 bp for forward reads and 150 for reverse reads to remove low-quality nucleotides. Chimeras were detected and removed using the default consensus method in the dada2 pipeline. Then, ASVs with length between 300 bp and 360 bp were kept and deemed as high-quality ASVs. Taxonomy of the resultant ASVs was assigned to the genus level using the RDP classifier (v2.13) with a minimum bootstrap confidence score of 80. Species-level classification can be provided using blastn (v2.13.0) and refseq_rna database (updated 2022–06-10). Sequencing data was registered with NCBI Bioproject ID: PRJNA866398. At 2 weeks of age, animal number in each treatment group is SPF (n = 5, two females and three males from two litters), LPS (n = 5, two females and three males from two litters), Reuteri (n = 5, four females and one male from two litters), and LPS/Reuteri (n = 5, two females and three males from two litters). At 12 weeks of age, animal number in each treatment group is SPF (n = 5, one female and four males from two litters), LPS (n = 5, three females and two males from two litters), Reuteri (n = 5, one female and four males from two litters), and LPS/Reuteri (n = 5, three females and two males from two litters).

## Metabolomic analysis

### Data collection

Serum and brain samples were submitted to the Microbiome Metagenomics Facility of the DFI for metabolite extraction. Samples were analyzed on a Thermo Fisher liquid chromatography system coupled to an Orbitrap IQ-X mass spectrometer, operating in positive mode. 3 µL of sample was injected onto a Cortecs© UPLC T3 Column (1.2 µm, 2.1 × 100 mm) fitted with Cortecs© UPLC T3 guard at 30°C. The mobile phase A was water with 0.1% Formic Acid, and mobile phase B was 95% Acetonitrile with 0.1% Formic Acid. Gradient elution started with 0% B with a flow rate of 0.48 mL/min for 0.2 min and linearly increased to 97% B over 5 min, and these conditions were held constant for 1.0 min. Finally, re-equilibration at 0% B was performed for 1.5 min. The electrospray ionization conditions were set with the spray voltage at 3.4 kV, vaporizer temp at 400°C, and detection window set to 100–2000 m/z. Precursor selection for MS^2^ scans was set to 150–2000 m/z with dynamic exclusion after 2 times within 10 seconds. The isolation window was 1.5 m/z with no offset and a fixed collision energy of 30%. At 2 weeks of age, animal number in each treatment group for serum samples is SPF (n = 3, two females and one male from three litters), LPS (n = 3, two females and one male from two litters), Reuteri (n = 3, two females and one male from two litters), and LPS/Reuteri (n = 3, two females and one male from one litter). At 12 weeks of age for serum samples, animal number in each treatment group is SPF (n = 4, two females and two males from two litters), LPS (n = 4, two females and two males from three litters), Reuteri (n = 4, two females and two males from two litters), and LPS/Reuteri (n = 4, two females and two males from two litters). At 2 weeks of age, animal number in each treatment group for brain samples is SPF (n = 3, one female and two males from two litters), LPS (n = 3, three females from two litters), Reuteri (n = 3, two females and one male from two litters), and LPS/Reuteri (n = 3, one female and two males from one litter). At 12 weeks of age for brain samples, animal number in each treatment group is SPF (n = 4, two females and two males from two litters), LPS (n = 4, two females and two males from three litters), Reuteri (n = 4, two females and two males from two litters), and LPS/Reuteri (n = 4, two females and two males from two litters).

### Data processing

Raw data files were converted into open-source file format and processed using MZmine2 and the Feature-Based Molecular Networking function in the Global Natural Products Social Molecular Networking (GNPS) environment to identify features and match data to publicly available library spectra. MetaboAnalyst was used for statistical analysis and visualizations.

### MZmine

MZmine 2.53^74^ was used to create feature lists with abundances in each sample from the raw data. Settings used were based on manual inspection of the raw data for values that represented signals above the inherent noise level, typical peak shapes, and mass and retention time (RT) tolerances. First, a mass detection was used with a noise cutoff filter for both MS1 and MS2 scans to create a mass list for each data file. The Centroid mass detector was set to a level of 6.0E3 for the MS2 level and 1.0E4 for the MS1 level. The ADAP chromatogram builder algorithm was used to create extracted ion chromatograms at the MS1 level with a minimum group size of three scans, group intensity threshold of 1.0E4, minimum highest intensity of 3.0E4, and m/z tolerance of 0.015 Da or 5.0 ppm. Chromatogram deconvolution was performed using the Wavelets (ADAP) algorithm with an S/N threshold of 10, minimum feature height of 5E5, coefficient/area threshold 110, peak duration range of 0.00–1.00, RT wavelet range of 0.01–0.25, median m/z center calculation, m/z range for MS2 scan pairing 0.01 Da, RT range for MS2 scan pairing 0.1 min. The isotopic peak grouper module was used to group features that are isotopes with m/z tolerance of 0.01 Da or 5.0 ppm, RT 0.1 min, a maximum charge of 4, and the lowest m/z as representative isotope. A master feature list was created using the Join aligner module with m/z tolerance of 0.01 Da or 5.0 ppm, RT 0.1 min, and weight for m/z and RT set to be equal (1). The resulting feature list was filtered to remove duplicate features and then the gap-filling algorithm was used to fill in any missing values for peaks that were not detected with the previous algorithms. The peak-finder gap filling algorithm was used with intensity tolerance 10%, m/z tolerance 0.02 Da or 5.0 ppm, and RT tolerance 0.1 min. Peaks were filtered to remove any with a peak area less than 3.0E4. The resulting feature list was exported for GNPS analysis.

### The global natural product social molecular networking (GNPS)

A molecular network was created with the Feature-Based Molecular Networking (FBMN) workflow^75^ on GNPS (https://gnps.ucsd.edu)^76^. The results from MZmine2 were exported to GNPS for FBMN analysis. The data were filtered by removing all MS/MS fragment ions within ±17 Da of the precursor m/z. MS/MS spectra were window filtered by choosing only the top six fragment ions in the ±50 Da window throughout the spectrum. The precursor ion mass tolerance was set to 0.02 Da and the MS/MS fragment ion tolerance to 0.02 Da. A molecular network was then created where edges were filtered to have a cosine score above 0.7 and more than 6 matched peaks. Further, edges between two nodes were kept in the network if and only if each of the nodes appeared in each other respective top 10 most similar nodes. Finally, the maximum size of a molecular family was set to 100, and the lowest scoring edges were removed from molecular families until the molecular family size was below this threshold. The analogue search mode was used by searching against MS/MS spectra with a maximum difference of 100.0 in the precursor ion value. The library spectra were filtered in the same manner as the input data. All matches kept between network spectra and library spectra were required to have a score above 0.7 and at least 6 matched peaks. The job can be accessed at https://gnps.ucsd.edu/ProteoSAFe/status.jsp?task=95ff61735c414baabd25a8fc0aeb7888.

### Feature list filtering

The feature list from GNPS was exported with putative IDs associated and filtered in Excel using peak areas found in blanks and quality control injections to remove low-quality features from the statistical analysis. To filter out blank peaks, first any features with raw peak area of over 1E6 found in any solvent blank sample were removed. Features within a sample were then normalized by dividing peak area by the peak area of the cholic acid internal standard in that sample. Features with normalized peak areas in samples less than or equal to the peak areas found in method blank controls were removed. A pooled QC of each sample treatment group was injected throughout the run, and three injections of each QC were used to calculate the percent coefficient of variation (%CV) of features. Any features with normalized peak area %CV of greater than 10% across the three pooled QC injections were eliminated. Finally, features that GNPS matched known mass spectrometry contaminants and polyether polymers were removed, resulting in a list of 389 features.

### MetaboAnalyst

MetaboAnalyst^77^ was used to statistically analyze and visualize the feature list exported from GNPS and filtered down to 389 high-quality features. For each subset, any features that had a single or constant value across all samples were removed. Figures were exported from MetaboAnalyst.

## Statistics

16S rRNA sequencing and metabolomic data analysis are stated in their respective sections above. All other data are presented as mean ± standard error of the mean (SEM). GraphPad’s Prism 9 (La Jolla, CA) software was used to perform statistical analyses. One-way ANOVA with Tukey’s multiple comparison *post hoc* test was used to determine differences among multiple groups. A *p*-value of <0.05 was considered significant.

## Supplementary Material

Supplemental Material

## Data Availability

Data are available from the corresponding author on request. https://www.ncbi.nlm.nih.gov/bioproject/PRJNA866398
